# Advanced Dielectric Resonator Antenna Technology for 5G and 6G Applications

**DOI:** 10.3390/s24051413

**Published:** 2024-02-22

**Authors:** Yingqi Zhang, Stanislav Ogurtsov, Vasilii Vasilev, Ahmed A. Kishk, Diego Caratelli

**Affiliations:** 1 The Antenna Company, 5656 AE Eindhoven, The Netherlands; stanislav.ogurtsov@antennacompany.com (S.O.); vasilii.vasilev@antennacompany.com (V.V.); diego.caratelli@antennacompany.com (D.C.); 2 Department of Electrical Engineering, Chalmers University of Technology, 412 96 Gothenburg, Sweden; ahmed.kishk@concordia.ca; 3 Department of Electrical Engineering, Eindhoven University of Technology, 5600 MB Eindhoven, The Netherlands; 4 Department of Electrical and Computer Engineering, Concordia University, Montreal, QC H3G 1M8, Canada

**Keywords:** dielectric resonator antennas (DRAs), DRA arrays, millimeter-wave bands, 5G, beyond 5G

## Abstract

We review dielectric resonator antenna (DRA) designs. This review examines recent advancements across several categories, specifically focusing on their applicability in array configurations for millimeter-wave (mmW) bands, particularly in the context of 5G and beyond 5G applications. Notably, the off-chip DRA designs, including in-substrate and compact DRAs, have gained prominence in recent years. This surge in popularity can be attributed to the rapid development of cost-effective multilayer laminate manufacturing techniques, such as printed circuit boards (PCBs) and low-temperature co-fired ceramic (LTCC). Furthermore, there is a growing demand for DRAs with beam-steering, dual-band functions, and on-chip alignment availability, as they offer versatile alternatives to traditional lossy printed antennas. DRAs exhibit distinct advantages of lower conductive losses and greater flexibility in shapes and materials. We discuss and compare the performances of different DRA designs, considering their material usage, manufacturing feasibility, overall performance, and applications. By exploring the pros and cons of these diverse DRA designs, this review provides valuable insights for researchers in the field.

## 1. Introduction

The early success of fifth-generation (5G) communication testbeds and commercial networks has spurred significant research in intelligent communication systems operating at frequencies below 6 GHz and in millimeter-wave (mmW) bands. Today, as we look to the future of wireless communication beyond 5G, the aim is to achieve extremely high data rates over distances ≥1 km [1,2,3]. Research demands focus on a wide range of applications, such as autonomous driving, the Internet of Things, wireless communication, and radio astronomy. The use of available wide-band mmW and sub-terahertz (THz) frequencies shows promise for delivering extremely high data rates of around 100 gigabits per second (Gbps). In this context, mmW antenna and array systems with features of high gain and efficiency, intelligent beamforming, diverse polarization, and wide or multiple frequency band support are seen as key technologies for the next generation of telecommunication systems [4]. These technologies can compensate for the considerable free space path loss, material losses, and reduced power generation ability of active electronic devices as we move to higher frequencies.

Typical front-end antenna solutions use printed circuit board (PCB) technology and various multilayer laminate-based packaging approaches [5]. While antennas like patch antennas and dipoles can be easily scaled up into large arrays at lower frequencies (microwave and lower mmW bands), challenges arise at higher mmW bands, where the size of the antennas approaches that of integrated circuits (ICs). This makes integrating the antenna with radio frequency integrated circuits (RFICs) challenging due to manufacturing tolerances and space constraints. Additionally, for off-chip antennas, using substrates with higher dielectric constants ($\epsilon_{r}$), like HDI ($\epsilon_{r}$ = 2.8–4.6) or LTCC ($\epsilon_{r}$ = 5–7.8) [5], can lead to high insertion loss at higher frequencies, reducing antenna efficiency and causing additional signal loss in transmission lines. Moreover, on-chip antennas suffer from limited bandwidth (BW) and reduced efficiency due to the impact of lossy silicon substrates.

**Figure 1:**
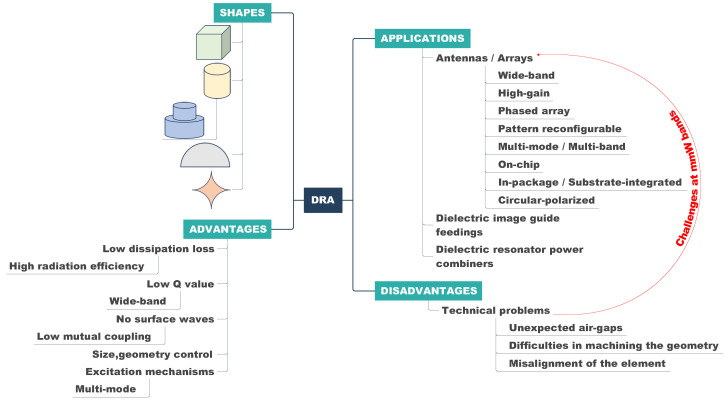
Performance, pros, and cons chart of the DRAs and their applications [6,7,8,9,10,11,12,13].

Conversely, dielectric resonator antennas (DRAs) present themselves as promising alternatives to printed, metal-stamped, and CNC-machined antennas, particularly in high-frequency applications such as mmW and beyond. The primary advantage of DRAs lies in their high efficiency without suffering from conduction losses. Traditionally, DRAs were constructed using ceramics characterized by high permittivity and quality factors ranging from 20 to 2000 [6,7]. In contemporary scenarios, more flexible materials like polyvinyl chloride (PVC) and PCBs have become viable options for manufacturing DRAs through machining, punching, 3D printing, or lithography techniques. The key benefits of DRAs include their compact size, wide BW, versatility in design, and polarization capabilities [8,9]. The size of a DRA scales to $\lambda_{0}/\sqrt{\epsilon_{r}}$ (where $\lambda_{0}$ denotes the free space wavelength at the resonant frequency $f_{0}$ and $\epsilon_{r}$ signifies the relative permittivity of the material constituting the radiating structure). By employing suitable excitation techniques, DRAs can be reshaped into various dimensions while maintaining the defined resonant frequency $f_{0}$, providing greater design flexibility (e.g., achieve a smaller footprint compared with printed antennas). Numerous studies have utilized a variety of DRA shapes, such as cylindrical, rectangular, (hemi-)spherical, and super shapes, to attain some characteristics such as a wide BW or high gain [10,11,12,13]. A performance chart of the DRAs and their applications are depicted in Figure 1.

Over the past few decades, DRAs have found widespread application across various domains, especially at microwave frequencies, primarily owing to the advantages mentioned earlier. In this paper, we review the latest advances and the design challenges associated with DRAs, with a particular focus on mmW antenna and array designs. The structure of this review is organized as follows:

- Section 2 provides a comprehensive overview of compact off-chip DRAs and their applications in array configurations.
- Section 3 introduces the concept of on-chip DRAs, which serve as gain directors in high mmW bands.
- Section 4 reviews the state-of-the-art beam-steerable DRAs designed to operate mostly in mmW bands.
- Section 5 reviews the dual-band DRAs in shared aperture designs up to mmW bands.

Finally, we conclude our review with a comprehensive discussion and a summary of our findings based on a visualized performance comparison of the various DRA implementations explored in the preceding sections.

## 2. Compact Off-Chip DRA and Array Designs toward Millimeter-Wave Frequencies

Traditional DRAs are fabricated as isolated volumes by 3D printing or machining techniques with hard ceramic materials and require additional accurate mounting on PCBs to cascade with feeds. Such a process becomes harder to implement in mmW frequencies, as the physical dimensions become smaller compared with standard fabrication and positioning tolerances. Meanwhile, DRAs feature outstanding performance at mmW or even terahertz frequency bands, thanks to their dielectric resonant modes with higher radiation efficiency and wider BW compared with printed antennas that suffer from conductor losses. However, the above challenges limit the applicability of DRA technology to high-power systems. Hence, researchers have employed various methods to manufacture small DRAs or DRA arrays for higher frequencies and co-fabricate them with the feeding networks.

**Figure 2:**
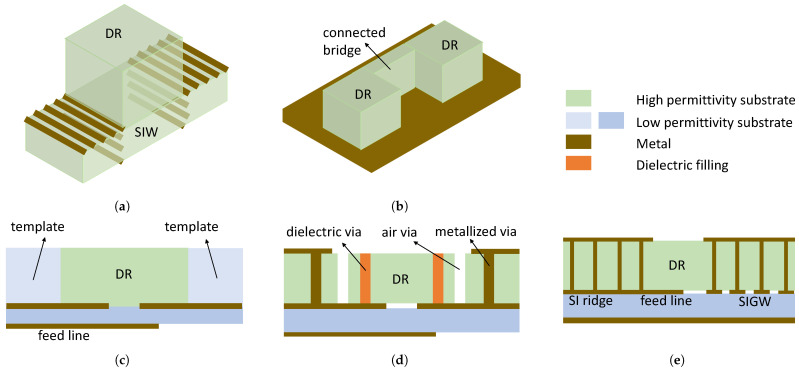
Co-fabrication methods for DR antennas and arrays: (**a**) SIW-backed DRA [14,15,16]; (**b**) connected DRA [17,18,19,20]; (**c**) in-template DRA [21,22]; (**d**) substrate-integrated DRA (SIDRA) [23,24,25,26,27,28,29]; (**e**) substrate-integrated gap waveguide DRA (SIGW DRA) [30,31].

**Figure 3:**
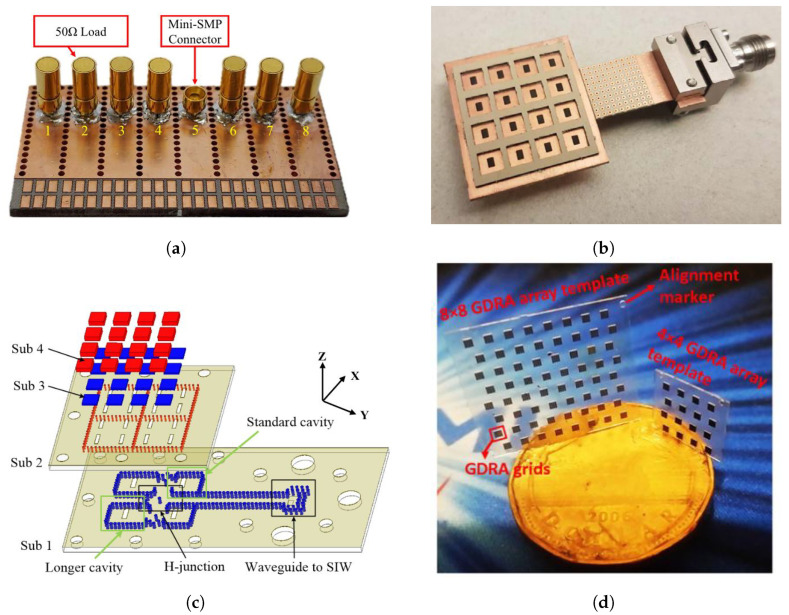
Examples of mmW co-fabricated and in-template DRA array architectures: (**a**) 28 GHz co-fabricated metasurface DRA array [16]; (**b**) 60 GHz connected DRA array [19]; (**c**) 67 GHz in-template DRA array [32]; (**d**) 60 GHz in-template XRL DRA array [21].

**Figure 4:**
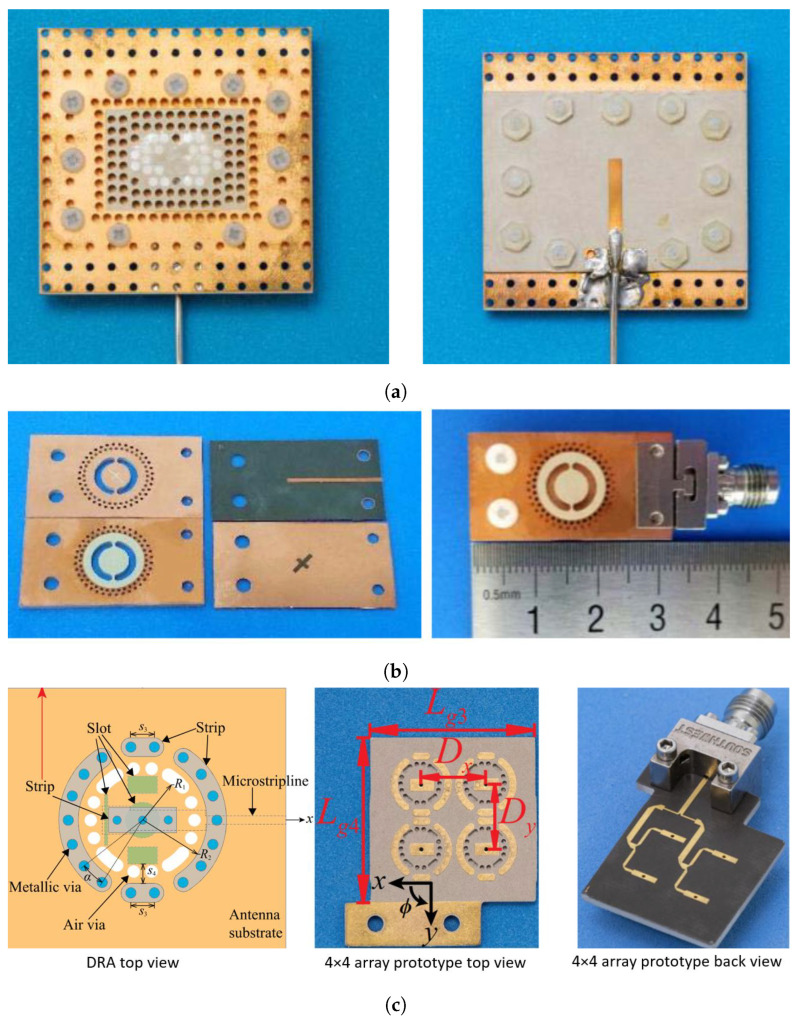
Examples of SIDRA configurations: (**a**) 9 GHz wideband SIDRA prototype top and back views [24]; (**b**) 24 GHz circular polarized SIDRA [27]; (**c**) 28 GHz filtering SIDRA [28].

These co-fabrication methods are illustrated in Figure 2: (1) manufacture the DRAs and feed jointly in one substrate (see Figure 2a); (2) connect each DRA element with dielectric bridges so that the array becomes one connected body, as shown in Figure 2b; (3) mount each DRA element in a low-permittivity template to enhance the alignment accuracy with the feeding network, as depicted in Figure 2c; (4) substrate-integrated DRAs in metal cavities (SIDRAs) with drilled vias or dielectric-filled vias to excite the desired DR modes (see Figure 2d); and (5) SIDRAs realized in substrate-integrated gap waveguide (SIGW) technology, where the feeding network closely affects the DRA modes (see Figure 2e). Recent microwave and mmW compact DRA and DRA array designs based on the above methods are cross-compared in Table 1 and introduced below.

### 2.1. Co-Fabricated and In-Template DRAs

When stitching DRA array elements with the PCB containing the feeding network, alignment problems can arise. To avoid these issues, end-fire DRAs can be co-fabricated with the feeding network on a single substrate. In 2016, Chu et al. co-fabricated a rectangular 1×4 DRA array with an SIW feeding network on one common PCB [14], as shown in Figure 2a. However, this design has limited design versatility and BW. In 2021, Omar et al. designed a beam-steerable cavity-backed DRA and its 1×4 array in low-temperature co-fired ceramic (LTCC) technology [15]. This design offers a wide BW of 47.1% thanks to its customized feeding structure. In 2022, Federico et al. adopted a similar DRA array solution with wide-angle beam-steering performance by integrating a metasurface with the DRs [16] as it appears in Figure 3a. This co-fabricated DRA array is possibly the most compact array topology, comprising DRAs and the relevant feeding network in the same technology. However, this solution cannot be easily applied to planar array configurations due to the bulky SIW feeding network.

More commonly, DRAs are designed using high-permittivity ($\epsilon_{r}$) materials, whereas the relevant feeding network is designed in microstrip or stripline technology (see Figure 2b). In such cases, alignment between the DRAs and the feeds is crucial to ensure the desired performance. In [17], Liu et al. fabricated DRA elements with connected dielectric bridges in a 1 × 4 array operating at 26 GHz. The DRAs are stitched onto the PCB with the integrated feeding network. Although significant BW (20%) and efficiency (91%) were achieved, this design approach is still subject to challenges regarding alignment tolerances and manufacturing complexity when applied to higher frequencies or large planar array structures. A similar approach was adopted to 60 GHz in [18], where a 4×4 cylindrical connected DRA array was fabricated by drilling out an unneeded PCB area. However, as the DRA size becomes smaller at higher frequencies, such a topology becomes prone to reduced mechanical reliability. In [19], the authors designed the DRA element in a rectangular ring shape so that the 4 × 4 array was a connected grid shape and manufactured in one PCB laminate, as Figure 3b shows. The ring-shaped DRA provides symmetric radiation characteristics as a magnetoelectric dipole but with a higher gain of 21 dBi at 60 GHz.

Mounting DRAs in low-permittivity material templates is a cost-effective way to accurately place the elements. However, manufacturing tolerances at mmW frequencies pose a challenge due to the small element sizes. A rectangular DRA side length can vary from 0.2 to 0.4 times the free space wavelength or 0.25$\lambda_{0}$ at 25 GHz or 60 GHz (namely 3 mm or 1.25 mm, respectively), which makes fabricating such radiating elements challenging and expensive on ceramic or silicon-based substrates. To tackle this problem at high frequencies, Chen et al. enlarged the rectangular DRA side length from 0.22λ to 0.4λ (that is, 1.8 mm) at 67 GHz by stacking two different substrates and exciting them with high-pass filtering SIW feeds [32], as shown in Figure 3c. As the lower cutoff frequency of the DRA is limited by the SIW, the DR’s fundamental mode only resonates in an effective area of $0.22\lambda^{2}$. The stacked substrates contribute to exciting the $TE_{112}^{x}$ mode as well, improving the BW (up to 16.4%) and enhancing the radiation efficiency above 72% for the 4×4 array configuration.

There is an alternative method for realizing 3D uniform DRA cubes called deep X-ray lithography (XRL). With this technique, the side lengths of DRA elements (which are typically around 1 mm) can be structured in photoresist material and ceramics [21,22], allowing for better manufacturing quality. This method has been successful in producing DRA arrays up to 60 GHz. In 2017, Qureshi et al. created a monolithic polymer-based DRA array using acrylic templates at 60 GHz. The array had an impedance BW of 12% and a radiation efficiency of over 90%. In 2019, Mazhar et al. used XRL on a low-$\epsilon_{r}$ polymethyl methacrylate substrate with micro-sized metal grids (see Figure 3d). These grids resulted in an equivalent $\epsilon_{r}$ of approximately 22.4 and improved the radiation characteristics. The corresponding 8×8 array had a wide BW of 8% and a superior radiation efficiency of over 95%.

### 2.2. Compact Substrate-Integrated DRAs

To circumvent alignment issues, one approach involves utilizing a multilayer PCB process to construct DRA arrays with integrated feeds, resulting in substrate-integrated DRAs (SIDRAs). High-permittivity PCB laminates such as Rogers 6010 or 3010 [33] (with $\epsilon_{r}$ ≈ 10–13) are typically employed as carriers for DRAs. These are then combined with a second laminate of a lower $\epsilon_{r}$, which contains the printed feeding networks, as depicted in Figure 2d. In the past, dielectric-filled or perforated vias were employed to embed various equivalent $\epsilon_{r}$ within the dielectric material to reach equivalent boundary conditions for DRAs [34] or to realize a wide impedance BW [35]. These methods can be realized in PCB technology for mmW in-substrate DRA arrays as well.

In 2021, Kremer et al. [23,24] utilized vias filled with a high dielectric constant material in DRAs operating in the microwave frequency band (see Figure 4a). This approach allowed achieving an effective dielectric constant of approximately 15. By integrating both dielectric and air vias within the DRA cavity, they were able to excite multiple resonant modes, obtaining a BW ≥33% and a radiation efficiency exceeding 77%. This combined via technique enhances the versatility of DRA designs, offering greater design freedom while maintaining a relatively low profile of ≤0.1$\lambda_{0}$. However, this comes at the cost of an increased antenna size, which typically ranges from 0.6 to 1.2$\lambda_{0}$ in the side length. Additionally, the use of filling vias with diameters around 1 mm may restrict their application at higher millimeter-wave (mmW) bands. Nonetheless, SIDRA designs hold promise for mmW-band applications due to their adaptability. Various functionalities have been explored in SIDRA implementations up to 60 GHz, utilizing air and metallic vias. Reported works demonstrate capabilities such as dual-polarization [26], circular-polarization [25,29], filtering response [28], and beam-steering performance [36].

In Figure 4b, Yang et al. presented a circularly polarized SIDRA at 24 GHz using air vias, air cavities, and metal vias in PCB technology [27]. Circular polarization is achieved through a straightforward cross-feeding slot mechanism. The introduction of a cylindrical ring-shaped air cavity excites both the fundamental and higher-order $HEM_{11\delta }$ and $HEM_{12\delta }$ modes, which contribute to a stable gain across the operating BW and enable a wide axial ratio (AR) BW of 30%. By applying a similar methodology, a 2×2 array was designed for operation at 60 GHz using metallic via fences to ensure higher isolation among the unit cells [29]. In Figure 4c, Liu et al. adapted the described design methodology by combining air and metal vias with connecting strips to excite multi-mode resonances while implementing radiation cancellation at unwanted frequency bands to achieve a filtering response. Filtering dielectric resonator antennas (DRAs) have been proposed initially at microwave frequencies [37]. However, this is the first report of such technology in the mmW band with a compact and relatively simple radiating structure featuring an outstanding in-band radiation efficiency above 72%.

In [30,31], Ma et al. proposed a new concept of SIDRA surrounded by substrate-integrated gap waveguide (SIGW) structures, as depicted in Figure 2e. In [30], the authors proposed SIGW DRA cavities fed by microstrip lines on two-layer PCB laminates. These cavities consist of substrate-integrated electromagnetic bandgap (EBG) structures. The resulting SIGW DRAs are not only compact but also fabricated in multilayer PCBs without the need for air- or dielectric-filled perforations or supplementary assembly processes. The reduced size of these SIGW DRAs also offers advantages in terms of array integration [31].

## 3. mmW On-Chip DRAs

In the domain of standard integrated circuit (IC) technologies, on-chip antennas such as slots or dipoles implemented within the silicon or GaA substrate commonly encounter the challenge of low radiation efficiency. This problem arises due to large energy dissipation in the high-$\epsilon_{r}$ substrate. Conversely, DRAs have higher Q values in high-$\epsilon_{r}$ materials. In [38], a cylindrical DRA with its feeding network was micromachined in a single silicon wafer. Remarkably, this DRA achieved 7 dBi gain with a radiation efficiency of 79.35% at 60 GHz. Another classification of on-chip antennas involves designs using an additional film or substrate on top of the slot or launcher on the back end of the line (BEOL), which allows more design flexibility. However, at mmW frequencies, these on-chip antennas (especially printed antennas) still exhibit poor efficiency, and they require a substantial chip area. The main challenge in developing on-chip antennas that display broadband and broadside radiation lies in the inherent limitations posed by the thickness of the available on-chip substrate, as noted in prior research studies [39,40,41,42,43]. In contrast, on-chip DRAs offer a wide range of material options and can act as a radiation director to enhance the broadside gain (Table 2).

### 3.1. On-Chip Spherical Self-Aligning DRAs

To address the aforementioned alignment and integration challenges, spherical DRAs have been designed for integration at the BEOL with self-alignment capabilities [52]. Since 2016, Hesselbarth et al. from the University of Stuttgart investigated the characteristics of various resonant modes of a dielectric sphere and, based on such studies, proposed a series of self-aligning on-chip DRA designs that cover frequencies ranging from the V band to the D band.

**Figure 5:**
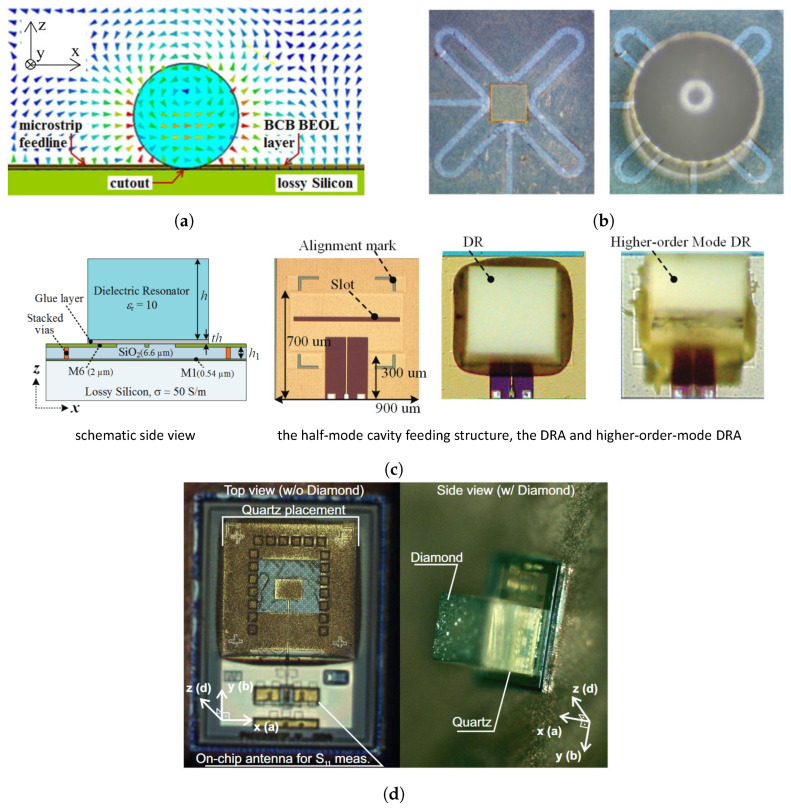
Examples of on-chip DRA designs: (**a**) cross-sectional view of a spherical DRA and the stripline feed. E-field of $TE_{1\delta 3}^{y}$ is depicted [44]; (**b**) 105 GHz loop fed self-aligned spherical DRA prototype view [44]; (**c**) 135 GHz on-chip high-gain RDRA [48]; (**d**) 400 GHz on-chip stacked RDRA [43].

As Figure 5a,b depict, the spherical DRA is positioned at the BEOL and is excited using an on-chip microstrip coupling technique. To ensure precise alignment, a shallow crater is etched into the polymer layers of the BEOL atop the semiconductor in close proximity to the microstrip feed. When a spherical DRA is placed within this crater, it naturally achieves highly accurate positioning. The DRA shown in Figure 5a was fabricated from alumina with a relative permittivity of 10 and a loss tangent of 0.0005. In [44], a microstrip loop resonator was used to excite a dual-polarized spherical DRA operating at 108 GHz. The measured BW was 9%, and the feed-port isolation was 20 dB. The measured gain ranged from 7 to 9 dBi, while the simulated efficiency peaked at 80%.

The study presented in [53] suggests that the dominant modes $E_{101}$ and $H_{101}$ are more effective for spherical DRAs compared with other modes. This assertion is supported by both field simulations and experimental measurements. In the proposed design, a microstrip line integrated into a thin circuit board is used to excite a microstrip resonator. The fringing electric fields at the open end are responsible for stimulating a variation of the $E_{101}$ mode within the dielectric sphere, as illustrated in Figure 5b. This spherical DRA displayed a fractional BW of about 11% at 180 GHz and achieved a notable gain of 7.9 dBi, along with a radiation efficiency of 80%. These metrics signify a substantial enhancement in performance relative to other on-chip DRA and printed antenna configurations.

To further enhance directivity, researchers have employed a superstrate structure over the DRA, achieving a gain of 17.8 dBi in the V band and 18.4 dBi in the W band. However, it is important to note that the introduction of a superstrate or lens may also increase the on-chip footprint size [45,46].

### 3.2. On-Chip DRAs on BEOL

DRAs are commonly mounted on the BEOL of a chip and excited through a slot, dipole, or antenna launcher designed on the BEOL. Usually, due to the dissipation of the energy in the lossy silicon substrate, on-chip DRAs working at fundamental resonating modes have a limited gain (1 dB at 27.8 GHz) and BW [54]. In 2012, Hou et al. developed techniques for fabricating on-chip DRAs that operate at 130 GHz using CMOS technology [47]. To improve the antenna gain and efficiency, two DRAs were stacked above the on-chip feeding structure. These stacked DRAs achieved a fractional BW of 11% and a measured gain of 4.7 dBi, albeit with an efficiency of just 43%. In 2014, Hou et al. presented cube-shaped DRAs realized through a standard 18 μm CMOS process. The proposed DRAs operated on higher-order modes in order to enhance the gain [48], as shown in Figure 5c. A DRA designed with a half-mode backed cavity as the feeding structure and operating in the $TE_{\delta 11}^{x}$ mode reached a measured gain of 3.7 dBi at 132 GHz, with a radiation efficiency of 62%. Other high-order mode DRAs exhibited higher gains, with 6.2 dBi for the $TE_{\delta 13}^{x}$ mode and 7.5 dBi for the $TE_{\delta 15}^{x}$ mode.

These on-chip DRA designs were further corroborated by the authors in 2×1 and 4×1 arrays [49]. The DRA arrays, incorporating the feeding networks into a 6.6 μm SiO_2_ substrate yielded gains of 6.3 dBi and 7 dBi in the 2×1 configuration and 7 dBi and 8.2 dBi in the 4×1 configuration for the $TE_{\delta 11}^{x}$/$TE_{\delta 13}^{x}$ modes. This empirical evidence is critical for the future design of on-chip antenna arrays, where the maximum gain and chip area considerations are paramount.

An additional application of higher-order mode DRAs was demonstrated with 0.1 μm GaA pHEMT technology at 270 GHz, where an on-chip cavity-backed patch antenna excited the $TE_{\delta 13}^{x}$ mode of the DRA [50]. This on-chip DRA delivered a numerically simulated gain of 6.4 dBi and a radiation efficiency of 75%.

As on-chip DRA designs progress to the (sub-)terahertz frequencies, accurately manufacturing small DRAs that display suitable gain and BW constitutes a formidable challenge. Li et al. realized an on-chip DRA of merely 400 μm by 300 μm by slicing a silicon wafer [51]. This DRA, manually attached to a 0.18 μm CMOS chip and operating in the $TE_{\delta 17}^{x}$ mode, showed a gain improvement of 6.7 dB over a standard on-chip patch antenna.

To enhance gain, techniques involving lens integration and high-order mode DRAs have been adopted, along with on-chip stacked DRAs comprising two different materials to improve the BW and gain. For instance, Deng et al. in 2015 presented a stacked DRA fed by a substrate-integrated waveguide (SIW)-backed patch at 340 GHz using a 0.13 μm SiGe BiCMOS process [42]. The upper DRA, made of high-permittivity material, was mechanically supported by a lower DRA with reduced $\epsilon_{r}$. This configuration achieved a peak gain of 10 dBi and a radiation efficiency of up to 80% at 340 GHz. Similarly, Gashi et al. proposed a 400 GHz on-chip stacked DRA fed by a metasurface-backed patch. This set-up included a supporting quartz layer and a diamond-based anti-reflection layer as a radiation director, as Figure 5d depicts. It showed an impedance BW of 25.6%, covering the frequency range from 340 GHz to 440 GHz with efficiency between 50% and 66% and directivity of up to 10.4 dBi, which could be increased up to 27 dBi through the integration of an additional lens [43].

## 4. Beam-Steerable DRA Arrays

Phased arrays have been utilized in a wide range of fields, from military applications to wireless communications. In traditional phased arrays, the selected antenna elements must be compact, meeting the dense inter-element spacing requirements (usually $\leq 0.5\lambda_{0}$ to prevent the appearance of grating lobes) while still maintaining a significant gain during scanning. Commonly used microstrip antenna-based solutions can typically achieve scan angles up to ±50° with a total scan loss of about 4–5 dB [55]. DRAs offer versatility in shape and size along with various resonant modes, providing more degrees of freedom during the design stage to achieve specific performance at the array level, such as wide-beam or wide-angle scanning.

For conventional PAA architectures, most designs aim to fulfill wide-beam capabilities [36,56,57,58,59,60,61], with their maximum scan range ($|\theta_{sc}|$) reaching up to 60°. Another design approach involves the use of pattern-reconfigurable antennas with active phase-shifting circuits embedded within each radiating element to enable beam shaping and achieve wide steering angles [62,63,64,65,66].

### 4.1. Wide-Beam DRAs

DRAs typically display a footprint size that is smaller than the grating lobe-free inter-element spacing of $\lambda_{0}/2$. They are generally manufactured as three-dimensional dielectric components, which provide the flexibility to incorporate additional structures between DRA elements in an array configuration to mitigate parasitic mutual coupling [67]. Traditionally shaped beam-steerable DRA arrays (Table 3) are demonstrated in isolated dielectric volumes and in-substrate compact array forms, where the steered beam can be realized by cascading phase-shifting circuitry or loading low-cost reactive loads to the feeds [34,68,69]. However, traditionally shaped DRAs usually have limited scanning angles because their fundamental resonating modes are not characterized by wide-beam radiation patterns. To achieve extensive scan ranges while preserving a large operating BW at the array level, various modifications can be implemented in such a way as to broaden the beamwidth of the embedded antenna elements. For example, the integration of parasitic elements and metasurfaces can be employed for this purpose.

**Figure 6:**
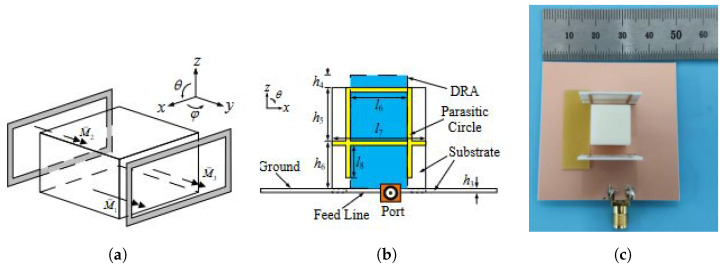
Example of wide-beam DRA design in [57]: (**a**) diagram of a DRA with two parasitic loops, (**b**) the schematic side view, and (**c**) the prototype of the proposed DRA topology with parasitic loops and strips.

In [57], Zhang et al. designed a planar 8 × 8 DRA array capable of a wide scanning range up to ±80° and ±75°) in the E and H planes, respectively, with a scan loss of no more than 4.5 dB. This was achieved by introducing parasitic vertical loop-like strips between the elements on the H plane. This method positions a pair of loops on each side of the radiation unit, inducing symmetrical horizontal magnetic currents (HMCs) in conjunction with the ground. By combining the HMCs from the DRA with those from the suspended pair, a broader beamwidth is obtained. This principle is illustrated in the design diagram in Figure 6a and is substantiated by the topologies shown in Figure 6b,c. The HMCs effectively expand the half-power beamwidth (HPBW) of the embedded element’s radiation pattern to up to 205° in the E plane and 140° in the H plane.

**Figure 7:**
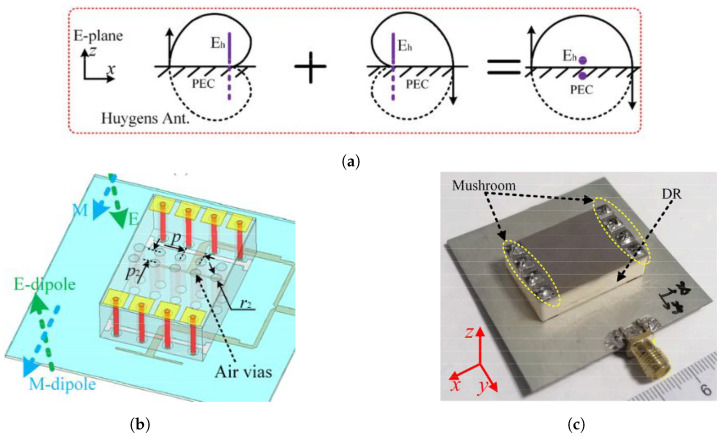
Example of the miniaturized mushroom-loaded wide-beam DRA in [58]: (**a**) basic principle of two out-of-phase Huygens sources for wide-beam operation and the (**b**) topology and (**c**) prototype of the DRA unit loaded with a pair of mushroom walls.

In another approach detailed in [58], Wang et al. introduced a wide beam technique involving a pair of miniaturized, equal-amplitude, and out-of-phase Huygens sources. This method increased the HPBW of the DRA from 90° to 194°. The adapted topology of the rectangular DRA is displayed in Figure 7. Mushroom structures are positioned on each side of the DRA, and the antenna is excited by dual slots, with air vias positioned centrally for separation. This configuration creates a miniaturized Huygens source that produces a tilted beam by merging the $TE_{111}$ mode of the DR (magnetic dipole) with the mode induced by the mushroom structures (electric dipole), as depicted in Figure 7a. The resulting planar array can scan up to ±60° and ±45° in the E and H planes, respectively, and supports a wide fractional BW of 12.8%.

**Figure 8:**
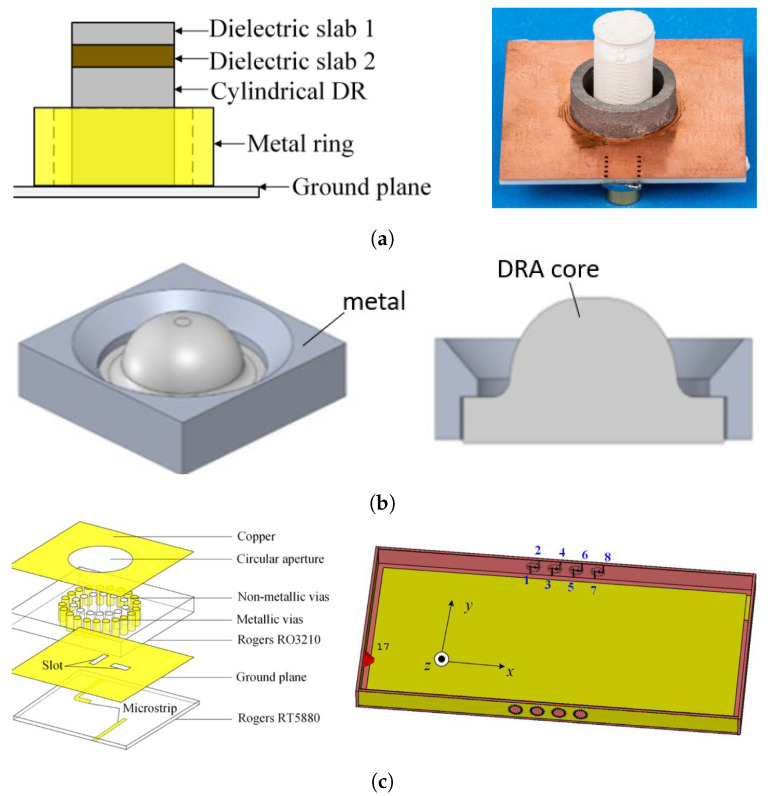
X-band and mmW DRAs for traditional electrical scanning PAAs: (**a**) 10.7 GHz DRA with two dielectric slabs and outer metal ring [59], (**b**) 3D view and cross-section of in-cavity DRA at 24 GHz ISM band [56], and (**c**) configuration of 27 GHz SIDRA for mmW mobile handset applications (1–8 are ports index) [36].

As the working frequency increases, implementing wide beam techniques through complex EM structures becomes impractical due to stringent manufacturing tolerances and reduced spacing between radiating elements. To address this aspect, simpler beam-shaping techniques have been developed for the *X* band and mmW bands. In [59], Su et al. optimized a cylindrical DRA design by mitigating the peak broadside gain and tailoring the beam in such a way as to encompass the desired scanning range. This was achieved by integrating a peripheral metal ring and positioning two dielectric slabs atop the DRA, as depicted in Figure 8a. In this design, the bottom base of the DRA continues to resonate at the dominant $HEM_{11\delta }$ mode, while the added dielectric slabs create non-uniform magnetic surfaces to shape the beam with a dual-peak distribution. The E-plane and H-plane beamwidths were verified to extend to 172° and 149°, respectively. A linear array in the H plane demonstrated a scan range of ±72° and a fractional BW of 14.9%, marking it as exceptional among the complex wide-beam array designs documented.

At mmW frequencies, the reduced inter-element spacing in traditional electrically steered arrays necessitates precision-engineered DRAs that are accurately aligned and assembled. This presents a significant challenge for wide-beam array designs, as evidenced by the scarcity of relevant research studies in the literature. In [56], Ogurtsov et al. showcased a 64 element DRA array design operating at 30 GHz with a fractional BW of 10% and a scan range of ±60° across the E, H, and D planes, which is notable as the first documented planar electrically scanned DRA array at the mmW bands. As shown in Figure 8b, each DR element consists of a cylindrical and a hemispherical DRA enclosed by an open metal cavity, which significantly mitigates mutual coupling and maintains a high radiation efficiency of ≥80% within a densely packed array lattice.

Other mmW beam-steering solutions utilize compact DRA structures, as discussed in the preceding section, which are advantageous for fabrication but inherently restrict their suitability for wide-beam applications [15,36,61]. For instance, the SIDRA shown in Figure 8c can only perform 1D beam-steering, limited by the inherent characteristics of the dominant DRA modes.

### 4.2. Pattern-Reconfigurable DRAs

Traditional PAA systems are fundamentally limited in scan range because of the finite spacing between elements. This design challenge has directed research toward hybrid beam-steerable arrays with pattern-reconfigurable antennas, promising enhanced steering capabilities beyond ±60°. However, this capability often comes at the cost of complex feeding or biasing networks [62,63,64,65,66,70]. Most pattern-reconfigurable DRAs reported to date operate at sub-mmW bands.

**Figure 9:**
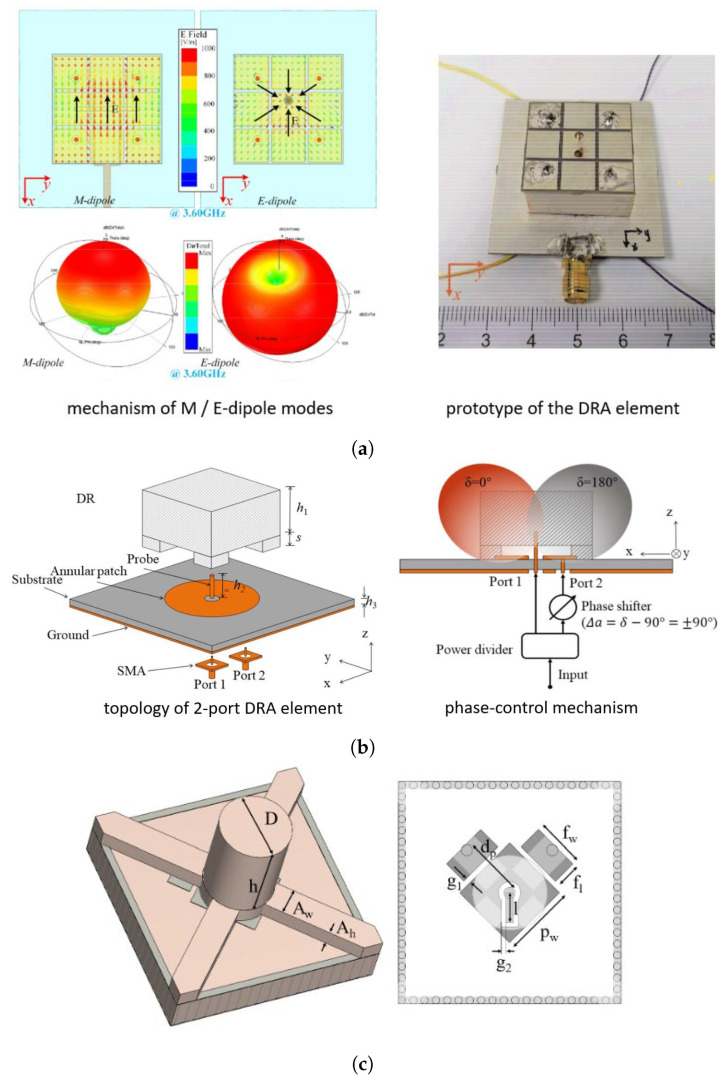
Examples of pattern-reconfigurable DRAs for beamsteering applications: (**a**) 3.65 GHz metasurface loaded DRA based on equivalent Huygen source design [62]; (**b**) 3 GHz phase-controllable DRA with titled beams [65]; and (**c**) 28 GHz simple switch beam DRA [66].

In [62], Wang et al. adapted a metasurface loaded DRA [58] for pattern reconfiguration by making use of PIN diodes. As illustrated in Figure 9a, different feeding elements excite the metasurface, enabling equivalent magnetic or electric dipole radiation modes. This approach reshapes the main beam from a broadside to a directional (θ=40°) radiation pattern. Notably, the proposed design is rather compact, with an overall size of $0.33\times 0.22\times 0.09\;\lambda_{0}^{3}$, and achieves a substantial scan range of ±70°. The authors also designed a planar array capable of covering scan ranges of (±60°, ±65°) in the E and H planes with a wide fractional BW of 12.8% using the same beam-steering mechanism [63]. Moreover, they demonstrated the metasurface-based pattern reconfigurability in a digitally coded linear DRA array for 2D scanning and in a phase-controlled DRA for 360° beam scanning [71,72].

In [65], Chen et al. implemented a conceptually similar approach by switching between electric and magnetic dipole-like radiation modes in DRAs. As shown in Figure 9b, they designed a standard rectangular DRA with a dual feeding structure; switching the feeding ports alters the resonant modes in the DRA from dominant $TM_{011}$ (E dipole-like) to $TE_{111}$ (M dipole-like), achieving a steered beam that covers ±66° when phase shifts are applied at Port 2. Their 1×4 array can cover a ±81° scan range with minimal gain fluctuation (only 1.25 dB), which is remarkable in comparison with conventional wide-scan microstrip antenna arrays. However, the narrow impedance BW (of about 3%) displayed by the considered design may limit its application.

In [66], a dual-mode cylindrical DRA (CDRA) was proposed, featuring a compact design that can be easily fabricated using conventional PCB manufacturing processes. Contrary to previous complex reconfigurable mechanisms, the radiation characteristics of the proposed CDRA structure are controlled through two separate ports by applying a suitable phase shift, as depicted in Figure 9c. This set-up produces two tilted beams, covering from −10° to 70° and −70° to 10°. The planar array provides a ±70° scan range in the horizontal plane and a scan range from −20° to 30° in the vertical plane, facilitated by the adoption of a triangular array grid.

## 5. Dual-Band DRAs up to the mmW Band

Dual-band antennas are invaluable in wireless communications and satellite and radar applications due to their ability to share an aperture, thus saving significant space compared with two separate antennas covering different frequency bands. DRAs are particularly well suited for dual-band operation because of their compact volume and the capability to support multi-mode resonance at various frequencies. Table 4 lists some of the recently reported dual-band DRAs that operate at millimeter-wave (mmW) frequencies. As highlighted before, DRAs designed for mmW frequencies must be both compact and easy to manufacture with currently available technologies.

To achieve dual-band functionality, one approach is to combine a DRA with another radiating structure sharing the same aperture. Alternatively, the multi-mode resonance capabilities of the DRA at different frequencies. In [73], Sun et al. proposed a stacked integrated DRA (SIDRA) positioned at the center of a hollow patch antenna. However, with this design, attaining a large frequency ratio, such as 24/5.2 GHz, presents challenges due to the different resonant modes of the two basic antenna structures, particularly when using a high-permittivity substrate. Cui et al. followed a similar design methodology but utilized a low-permittivity substrate for the printed antenna and inserted a high-$\epsilon_{r}$ slab at the center of the substrate [74]. The antennas are excited through an aperture slot and operate at 39 or 28 GHz with a fractional BW of 12% or 14%. The radiating element features dual polarization and is characterized by compact size, which facilitates a broad scanning range of ±40°/±50° in a linear array configuration suitable for 5G communications.

**Figure 10:**
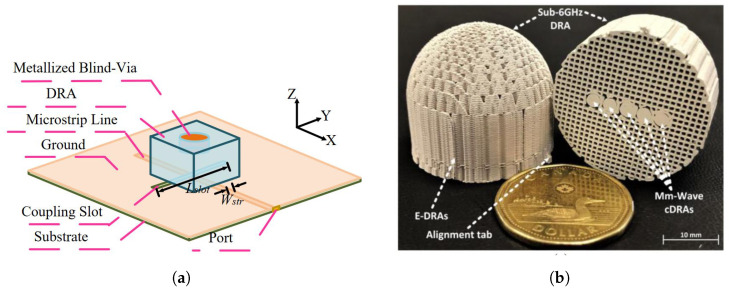
Configuration of the dual-band DRAs covering mmW bands: (**a**) PCB-based multi-mode DRA with metallic via [75] and (**b**) 3D-printed encapsulated DRA [76].

The most straightforward method for achieving dual-band operation is by exciting two distinct DR modes at considerably different frequencies. In Figure 10a, Wang et al. were able to excite a single rectangular DRA (RDRA) operating in $TEM_{131}$ and $TEM_{111}$ mode at 38 GHz and 16 GHz, respectively [75]. The design used only one metallic blind via in the PCB-based DRA to achieve multi-mode resonance. This compact design with a reduced size is promising for mmW array applications due to its ease of manufacturing.

Malfajani et al. suggested an alternative approach to develop multi-mode, dual-band DRAs through 3D printing technology, as shown in Figure 10b [76]. By adjusting the filling factor and therefore the density of the printed material, one can tailor the equivalent $\epsilon_{r}$ of the DRAs to resonate at selected frequencies. Smaller, denser cylindrical DRAs (CDRAs) were enclosed within a larger low-$\epsilon_{r}$ DRA, resulting in encapsulated DRAs. The larger DRA functions akin to a lens, enabling relatively high gain, while the smaller DRAs, arranged in a 1×5 array, contribute to enhanced gain and beam-steering capabilities. This design is compact and manufacturable, reaching a substantial frequency ratio of 30.5/3.6 GHz.

## 6. Discussion and Conclusions

**Figure 11:**
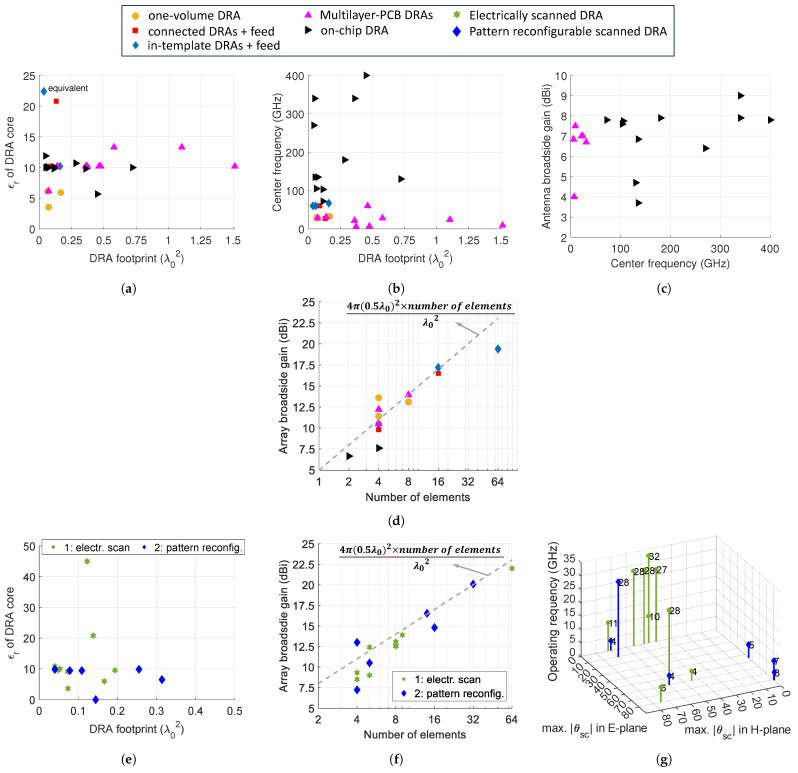
Performances of recent reported DRA designs discussed in this paper for compact DRA designs (off-chip and on-chip): (**a**) permittivity of the DRA core vs. the DRA footprint; (**b**) center operating frequency vs. DRA footprint; and broadside gains vs. center operating frequency for (**c**) single DRA designs and (**d**) array designs. For beam-steerable DRAs, (**e**) permittivity of the DRA core vs. the DRA footprint, (**f**) array broadside gains vs. number of elements, and (**g**) center operating frequency vs. maximum scan angles in 2D planes.

The performance metrics of various recently reported DRA designs (selected in this review), categorized in Table 1, Table 2 and Table 3, have been visualized in Figure 11. In the context of compact off-chip DRAs, as illustrated in Figure 11a and shown in Figure 11b, a considerable number of these designs were crafted with a small footprint size of ≤0.25$\lambda_{0}^{2}$. This compact footprint size is advantageous for array configurations. Alumina, characterized by a relative permittivity ($\epsilon_{r}$) of 9.8, is the most frequently chosen core material for DRAs due to its cost-effective manufacturing processes. Other ceramic materials with $\epsilon_{r}$ values ranging from 10 to 14 are also employed to ensure good quality (Q) values for the DRAs. On the other hand, PCB and LTCC technologies are not commonly used given their limited $\epsilon_{r}$ values, which are below seven. SIDRAs require complicated via punching and dielectric filling processes, which tend to enlarge the DRA footprint. Figure 11b provides a clear distribution of the different types of DRAs applied across the various frequency bands. Compact multilayer-PCB DRAs are commonly applied below 60 GHz, whereas on-chip DRAs cover the high mmW bands up to 400 GHz with relatively small footprints, largely due to their use of higher-order modes. It is important to note that in-template and connected DRA arrays can also be utilized up to 60 GHz, facilitated by high-precision machining (e.g., lithography [22]) and alignment techniques.

When comparing the achieved antenna gains for single-element designs across different frequencies, as displayed in Figure 11c, most SIDRAs achieve high gains exceeding 6.6 dBi, although their footprints are relatively larger. On the other hand, on-chip DRAs not only attain high gains in the range from 7 to 9 dBi but also operate effectively in high-mmW bands (∼400 GHz). However, it is worth noting that on-chip DRAs require the use of high-cost materials and machining techniques to ensure precise alignment on chips. These designs are not suitable for large array configurations due to limitations in high-cost chip area and alignment complexities. Typically, on-chip DRAs are combined with much larger and higher-profile lenses and superstrates to enhance their gain. In Figure 11d, we can observe the antenna gains for array configurations concerning the number of array elements. Clearly, most DRAs are high-gain and high-efficient. In-template DRAs and connected DRAs, used in arrays ranging from 16 to 64 elements, demonstrate promising manufacturing and assembly capabilities for large array configurations. Meanwhile, the potential of compact SIDRAs in large array set-ups is still being explored, with a focus on applying the appropriate manufacturing technology and improving the mechanical stability.

Figure 11e–g depict the performance characteristics of the beam-steerable antennas considered in this review. Conventional electronic scanning arrays require the inter-element spacing to be smaller than 0.5$\lambda_{0}$ to avoid grating lobes. Consequently, most reported DRAs are designed to resonate with high-permittivity materials (with $\epsilon_{r}\geq 10$) to minimize their footprints to less than or equal to $0.25\lambda_{0}^{2}$. Pattern reconfigurable antennas, which have switchable modes embedded in antennas or feedings, tend to have larger antenna sizes. Certain reconfigurable designs featuring elements loaded with mushroom structures or resonating in higher-order modes have successfully minimized their footprints ≤0.15$\lambda_{0}^{2}$ [62,63,70].

Figure 11f illustrates the array gains in relation to the number of elements. Most designs exhibit a 0.5–2 dB loss compared to the maximum available gains, calculated based on a square-latticed phased array spaced at 0.5$\lambda_{0}$. This discrepancy may arise from the introduced wide beam techniques or multi-mode schemes that bring additional insertion losses from extra structures and metallic losses. Nevertheless, some designs have achieved high gains with 32–64 elements [56,66]. Finally, Figure 11g presents the operating frequencies against the maximum scan angles in 2D planes for beam-steerable DRAs. Most designs exhibit outstanding scanning capabilities in the H planes due to the nature of fundamental mode resonances in DRAs. Most DRAs are primarily designed for 1D scanning, with only four demonstrating eligibility for achieving extensive scan ranges exceeding 60°. Only four designs are applied to 2D scanning, with just one design functioning at mmW bands [56]. The relative scarcity of mmW beam-steerable DRAs is due to the complexity of designing these antennas in large array configurations and enabling wide beam techniques at mmW frequencies. Therefore, advancing mmW beam-steerable DRAs continues to depend on the development of mature and accurate alignment techniques.

In this article, we conducted a comprehensive review of recent advancements in DRA designs spanning diverse categories. Our primary focus revolved around the utilization of DRAs in array configurations, particularly emphasizing their pertinence in mmW bands. For off-chip DRA designs, connected and in-template DRAs have emerged as cost-effective options, facilitating alignment within large array set-ups. Simultaneously, in-substrate DRAs, often achieved through technologies like PCB and LTCC, exhibit considerable promise due to their compact structures and the maturity of associated manufacturing techniques. However, it is imperative to acknowledge that via punching and dielectric filling may introduce challenges when applying these designs to higher frequencies. In the evolving landscape of 5G and beyond 5G communications, diverse applications such as radar, imaging, and the IoT have fostered a burgeoning demand for multifunctional antennas. DRAs with advanced capabilities, including beam steering, dual-band functionality, and on-chip alignment, have seen a notable surge in popularity. Their inherent flexibility, reduced conductive losses, and adaptability regarding shape and material choice render them compelling alternatives to conventional printed antennas. Through our critical comparison of various DRA designs, encompassing aspects of material selection, manufacturing feasibility, overall performance, and practical applicability, our insights serve as a valuable resource for the ongoing progress and development of this field.

## Figures and Tables

**Table 1:** Performances of compact off-chip DRAs.

	DRA Type	Feed and Polarization	Technology	$\epsilon_{r}$ of DRA	$f_{0}$ and BW (GHz, %)	Volume of DRA ($\times \lambda_{0}^{3}$)	Gain (dBi)	Rad. Eff. ^7^ (%)	Array and Spacing ($\times \lambda_{0}$)
[14]	R. DRA ^1^	SIW ^4^, LP, CP ^5^	1-layer PCB	6.15	30, 1	0.25 × 0.27 × 0.20	13.6 ^arr,M^	N/A	1 × 4 and 2 × 2, 0.85
[15]	R. DRA,	cavity backed, LP ^6^	LTCC	5.9	32.5, 47.1	0.37 × 0.45 × 0.17	11.4 ^arr,M^	70–85 ^t^	1 × 4, 0.4
[16]	R. DRA,	SIW, LP	1-layer PCB	3.55	28, 10	0.49 × 2.1 × 0.15	13.1 ^arr^	≥70 ^t^	1 × 8, 0.48
[17]	R. DRA rings, connected	slot, LP	glued on PCB	20.8	26.8, 20	0.30 × 0.44 × 0.07	∼9.8 ^arr,M^	91 ^ele^	1 × 4, 0.64
[19]	R. DRA rings, connected	slot, LP	glued on PCB	10.2	60, 13	0.99 × 1.1 × 0.25	∼21 ^arr,M^	88 ^arr^	4 × 4, 0.99, 1.1
[18]	C. DRA ^2^, connected	slot, LP	assemble 2 PCBs	10.2	60, 24	0.30 × 0.3 × 0.127	16.5 ^arr,M^	≥71%	4 × 4, 0.58
[32]	R. DRA stacked	SIW, LP	in-template, PCB	10.2 + 2.2	67, 16.4	0.40 × 0.40 × 0.18	17.2 ^arr,M^	72.3	4 × 4, 0.67
[22]	R. DRA	slot, LP	in-template, lithography	10	60, 12	0.24 × 0.24 × 0.12	10.5 ^arr,M^	≥90	2 × 2
[21]	R. DRA + artificial grid	SIW, LP	in-template, lithography	22.4 ^eq^	60, 8	0.20 × 0.18 × 0.10	15.2/19.4 ^arr,M^	73 ^arr,M^	4 × 4/8 × 8
[23]	R. SIDRA + air vias + dielectric vias	slot, dual LP	multilayer PCB	10.2	5.5, 35	0.61 × 0.61 × 0.09	6.84 ^M^	77	
[24]	R. SIDRA + air vias + dielectric vias	slot, LP	multilayer PCB	10.2	9, 33	1.25 × 1.21 × 0.11	6–8.7	∼92	
[25]	R. SIDRA + air vias + dielectric vias	slot, CP	multilayer PCB	10.2	6.8, 25.4	0.69 × 0.69 × 0.09	3.45–4.75	≥60	
[26]	R. SIDRA+ air vias + metal vias	slot, dual LP	multilayer PCB	10.2	21.7, 24	0.60 × 0.60 × 0.10	6–8	N/A	
[27]	C. SIDRA + air vias and cavity + metal vias	slot, CP	multilayer PCB	13.3	24, 35	1.05 × 1.05 × 0.12	6–8.15 ^M^		
[28]	C. SIDRA + air vias + metal vias + strips	slot, LP	multilayer PCB	13.3	28, 5	0.76 × 0.76 × 0.11	∼12.2 ^arr,M^	67 ^t^	2 × 2
[29]	C. SIDRA + air vias + metal vias	slot, CP	multilayer PCB	10.2	60, 12	0.68 × 0.68 × / ^e^	9–12 ^arr,M^	70 ^M,arr^	2 × 2
	R. SIDRA + SIGW ^3^ cavity	microstrip line, LP	multilayer PCB	10.2	31, 12	0.29 × 0.48 × 0.09	6–7.85	N/A	
[31]	R. SIDRA + SIGW ^3^ cavity	microstrip line, LP	multilayer PCB	6.15	28, 13.8	0.38 × 0.19 × 0.112	13.94 ^arr^	N/A	1 × 8, 0.56

^1^ Rectangular. ^2^ Cylindrical; radiation efficiency. ^3^ Substrate-integrated gap waveguide. ^4^ Substrate-integrated waveguide. ^5^ Circular polarization. ^6^ Linear polarization. ^7^ Radiation efficiency. ^arr^ Array. ^ele^ Element. ^eq^ Equivalent. ^M^ Measurement. ^t^ Total efficiency. ^e^ Estimated from figure.

**Table 2:** Performances of on-chip DRAs.

	Year	DRA Design Feature and Feed Structure	$\epsilon_{r}$	Chip Technology	f0 (GHz) and FBW (%)	DRA Size ($\times \lambda_{0}$)	Gain (dBi)	Rad. Eff. (%)
[44]	2018	spherical alumina DRA, self-aligns on cutout on BEOL	10.1	silicon-based	105, 8	D = 0.28 ^1^	7–8.5 ^M^	60–80
[45]	2020		10.7	/	180, 11	D = 0.6 ^1^	7.9 ^M^	80
[46]	2019	spherical DRA + partially reflective superstructure (PRS)	9.8	/	72, 5.6	DRA D = 0.38, superstrate D = 3.68 ^1^	7.9/17.8 ^wo/w,M^	70
			10.1	/	103, 9.1		7.6/18.4 ^wo/w,M^	70–80
[47]	2012	3 stacked RDRAs, coupled meandered slot	10, 2.4, 10	0.18 μm CMOS	130, 11	0.8 × 0.9 × 0.66	4.7 ^M^	43
[48]	2014	RDRA in $TE_{\delta 13}^{x}$, $TE_{\delta 15}^{x}$ mode, coupled slot	10	0.18 μm CMOS	135, 7	0.27 × 0.27 × 0.6–1	6.2–7.5 ^M^	46–42
		RDRA in half mode	10	0.18 μm CMOS	135, 13	0.27 × 0.27 × 0.1	3.7 ^M^	62
[49]	2017	RDRA in $TE_{\delta 11}^{x}$ mode, coupled slot	10	0.18 μm CMOS	135, 10	0.27 × 0.27 × 0.1	6.3/7 ^M,arr^	/
		RDRA in $TE_{\delta 13}^{x}$ mode, coupled slot	10	0.18 μm CMOS	135, 10	0.3 × 0.17 × 0.6	7/8.2 ^M,arr^	/
[50]	2018	RDRA, patch feed	/	0.1 μm GaAs pHEMT	270, 17	0.19 × 0.22 × 0.54	6.4	75
[51]	2017	silicon RDRA in $TE_{\delta 17}^{x}$ mode, patch feed	11.9	0.18 μm CMOS technology	340, 7.3	0.22 × 0.22 × 0.55	∼7.9	74
[42]	2015	stacked alumina RDRA, patch feed	9.8, 2.1	0.13 μm SiGe BiCMOS	340, 12	0.6 × 0.6 × 0.46	8–10 ^M^	65–80 ^M^
[43]	2022	AMC backed quartz RDRA + diamond layer + lens, patch feed	5.68, 3.75	1:35 nm mHEMT	400, 25.6	0.67 × 0.67 × 0.6 ^wo^	27 ^M^/7.8, ^D,wo/w^	50–66

^1^ diameter of sphere; ^M^ measurement; ^w/wo^ with/without superstrate/lens; ^arr^ array in 2 × 1 and 4 × 1; ^D^ directivity.

**Table 3:** Performances of beam-steerable DRA array.

	DRA Type	Feed and Polarization	Beam Steer Method	$\epsilon_{r}$ of DRA	$f_{0}$ and BW (GHz, %)	Volume of DRA ($\times \lambda_{0}^{3}$)	Array Broadside Gain (dBi) and Rad. Eff. (%)	Array and Spacing ($\times \lambda_{0}$)	E, H, or D Plane Scan Range (±,°)
[56]	CDRA + hemispherical DRA + metal cavity	LP	electro. scan.	9.5	28, 10	0.44 × 0.44 × 0.09	22 ^arr,e^, 80	8 × 8, 0.44	60, 60, 60
[57]	RDRA + parasitic strip walls	LP	electro. scan.	9.5	5.5, 5.5	0.22 × 0.22 × 0.44	12.5 ^2^, /	1 × 8 and 8 × 8, 0.4	80, 75, / ^3^
[58]	RDRA + metasurface	LP	electro. scan.	9.1	3.55, 12.8	0.33 × 0.22 × 0.09	9 ^4,M^, 81	1 × 5 and 4 × 4, 0.4	60, 45, /
[59]	CDRA stacked + outer ring	slot fed, LP	electro. scan.	10, 3, and 3.4	10.7, 14.9 ^M^	0.23 × 0.23 × 0.41	13.9 ^arr,M^, /	1 × 9, 0.45	/, 72, /
[60]	RDRA + parasitic DR strips	LP/CP	electro. scan.	45 and 69	10, 17	0.35 × 0.35 × 0.11	12.4 ^arr^, /	1 × 5, 0.5	/, 45, /
[61]	Ring RDRA + strip	LP	electro. scan.	20.8	26.9, 19.5	0.3 × 0.46 × 0.09	≥9.3 ^arr,M^, 89.6	1 × 4, 0.5	/, 40, /
[36]	CDRA + air vias + metal vias	DUAL LP	electro. scan.	10.8	27.5, 15	0.2 × 0.2 × 0.09	12.6 ^Mobi^, 95	2 × 1 × 4 ^Mobi^, 0.5	/, 28–138, /
[16]	R. DRA + metasurface	LP	electro. scan.	3.55	28, 10	0.49 × 2.1 × 0.15	13.1 ^arr^, ≥70 ^t^	1 × 8, 0.48	0, 55, /
[15]	SIW DRA	LP	electro. scan.	5.9	32.5, 47.1	0.37 × 0.45 × 0.17	6–11.4 ^arr,M^, 70–85 ^t^	1 × 4, 0.43	/, 45, /
[62]	RDRA + metasurface	LP	pattern-reconfig. ^1^	9.4	3.65, 14.1	0.28 × 0.28 × 0.11	10.5 ^M^, 78.4 ^M^	1 × 5, 0.5	/, 70, / ^M^
[63]	CDRA + metasurface	LP	pattern-reconfig.	9.4	3.5, 12.8	0.33 × 0.33 × 0.09	14.8 ^arr,M^, 77 ^M^	4 × 4, 0.46	60, 60, 75 ^M^
[64]	RDRA + ring-shape DRA	LP	pattern-reconfig.	9.8	4.9, 16.8 ^M^	0.5 × 0.51 × 0.28	13 ^e^, /	1 × 4, 0.95	15–60, /, /
[65]	RDRA + air gap	probe fed, LP	pattern-reconfig.	/	3, 3.3	0.38 × 0.38 × /	7.22 ^arr,M^, /	1 × 4, 0.5	0–81, /, /
[70]	RDRA	ring and probe fed, LP	pattern-reconfig.	9.8	7, 14	0.2 × 0.2 × /	16.53 ^arr^, 96	1 × 14, 0.46	80, /, /
[66]	CDRA + diagonal strip	patch fed, LP	pattern-reconfig.	6.45	28, 11	0.56 × 0.56 × /	20.1 ^arr^, 70 ^t^	8 × 4, 0.7, 0.56	20–30, 70, /

^e^ Estimated from figure. ^1^ Reconfigurable. ^2^ 1 × 8 array. ^3^ 8 × 8 array with 4.5 dB scan loss. ^arr^ Array. ^M^ Measurement. ^t^ Total efficiency. ^Mobi^ Array in mobile handset models in Figure 8c.

**Table 4:** Performances of dual-band DRAs covering mmW band.

	Topology	Feed and Polarization	Technology	$f_{0}$ and BW	$\epsilon_{r}$ of DRA	DR Modes	Volume ($\times \lambda_{0}^{3}$)	Gain (dBi)	Scan Angle
[73]	DRA + patch	Shared slot, LP	SIDRA	24, 3	10.2	$HEM_{11\delta }$		6.32	
			Printed patch	5.2, 2			0.36 × 0.36 × 0.027	3.93	
[74]	DRA + printed strip	Shared slot, dual LP	DR in PCB template	39, 12.56	45	$TEM_{111}$, $TEM_{131}$		6.4	±40°
			Printed strip, stub	28, 14.11	3.54		0.34 × 0.36 × 0.1	6.8	±50°
[75]	Multimode RDRA	Shared slot, LP	PCB + via	38, 9.7	3.55	$TEM_{131}$		14.2 ^1^	
				16, 35.3		$TEM_{111}$	0.32 × 0.32 × 0.2	10.6 ^1^	
[76]	Encapsulated DRA	Slots, LP	3D printing	30.5, 27	9			18 ^2^	±32°
				3.6, 33	4	$HEM_{11\delta }$	0.87 × 0.87 × 0.35	7.2	

^1^ 1×4 array gain. ^2^ 1×5 array gain.
